# Gene expression patterns of immune markers in rainbow trout during the early stages of seawater infections with *Piscirickettsia salmonis*

**DOI:** 10.3389/fvets.2025.1660383

**Published:** 2025-11-17

**Authors:** Héctor A. Levipan, Hernán Wicki, Fernanda Barrios-Henríquez, Francisco Pozo-Solar, Rute Irgang, Ruben Avendaño-Herrera

**Affiliations:** 1 Laboratorio de Ecopatología y Nanobiomateriales, Departamento de Ciencias y Geografía, Facultad de Ciencias Naturales y Exactas, Universidad de Playa Ancha, Valparaíso, Chile; 2 Laboratorio de Patología de Organismos Acuáticos y Biotecnología Acuícola, Facultad de Ciencias de la Vida, Universidad Andrés Bello, Viña del Mar, Chile; 3 Fund for Research Centers in Priority Areas (FONDAP), Interdisciplinary Center for Aquaculture Research (INCAR), Universidad Andrés Bello, Viña del Mar, Chile; 4 Laboratorio de Complejidad Microbiana y Ecología Funcional, Centro de Bioingeniería y Biotecnología, Universidad de Antofagasta, Antofagasta, Chile; 5 Centro de Investigación Marina Quintay (CIMARQ), Universidad Andrés Bello, Quintay, Chile

**Keywords:** piscirickettsiosis, salmon rickettsial septicemia, reverse transcription-quantitative PCR, immune gene marker, seawater bath challenge model, LF-89-like genotype, EM-90-like genotype, LF-89 genotype

## Abstract

Piscirickettsiosis is a fish disease caused by the facultative intracellular bacterium *Piscirickettsia salmonis*. This research aimed to study the immune response of rainbow trout (*Oncorhynchus mykiss*) during the first seawater farming stage upon infection with *P*. *salmonis*. Fish were challenged by immersion with *P*. *salmonis* type strain LF-89^T^ (genogroup 1) and the field isolates Psal-103 (LF-89-like genotype or genogroup 3) and Psal-104 (EM-90-like genotype or genogroup 4). A group of fish was treated with sterile AUSTRAL-SRS (Salmon Rickettsial Septicemia) medium. This group and fish from the infectious treatment and negative control were kept in a pilot-scale recirculating aquaculture system (RAS) for monitoring purposes. The *P*. *salmonis* load in trout skin and seawater was measured by reverse transcription-quantitative PCR (RT-qPCR) targeting an internal transcribed spacer (ITS) region. ITS transcripts were undetectable in trout skin samples before challenges and in trout skin from the sterile SRS medium treatment after challenges. The number of ITS transcripts in RAS seawater samples was 2.21 × 10^4^ ± 8.99 × 10^3^ copies per ng of total RNA at 15 days post-infection (dpi) and undetectable at 30 dpi. ITS transcript levels in trout skin were at maximum 15 dpi for all *P*. *salmonis*. For instance, the load of ITS transcripts in the skin of Psal-103-infected trout was 5.44 ± 2.58 × 10^6^ copies per ng of total RNA at 15 dpi. There were no significant differences in mortality between infection treatments. The cumulative mortality of trout from the negative control group was significantly lower than those from *P*. *salmonis*-infected trout. The expression of nine immunity-related genes was determined by RT-qPCR in the gills, spleen, liver, muscle, and head kidney tissues. An innate inflammatory response was associated with the expression of the *saa* and *tnf*-α genes in surviving fish. In addition, the downregulation of *oncmyk-dbb* and *IGHM* genes indicates that *P*. *salmonis* can interfere with the activation of CD4+ T cells and impair humoral immunity. Our findings suggest that the isolate Psal-104 has a higher immunogenic potential. Finally, our results support the use of non-lethal and non-invasive methods for analyzing fish skin as an early surveillance tool for piscirickettsiosis.

## Introduction

The facultative intracellular marine Gram-negative γ-proteobacteria *Piscirickettsia salmonis* is the etiological agent of piscirickettsiosis or salmon rickettsial septicemia (SRS). The disease was first reported in Chile in the 1980s and then during the next years in Canada, Scotland, Ireland, and Norway (1, 2), which is currently the world's largest salmon producer. Even though piscirickettsiosis does not causes significant economic losses in Norway and other salmon-producing countries, it was responsible for ~44.7% of deaths caused by infectious microorganisms in Chilean salmonid farms during 2023 (3). This disease causes economic losses exceeding US$1,000 million annually for the Chilean salmonid industry (4). Importantly, the disease affects rainbow trout (*Oncorhynchus mykiss*) during the ongrowing stage (3), which, in Chile, is carried out in a seawater farming stage. There are currently no effective treatments for piscirickettsiosis, other than antibiotics, with florfenicol being the drug of choice (5). The use of antimicrobial drugs in fish farming is a controversial issue due to the potential impacts on aquatic environments, risks of selecting and spreading drug-resistant bacteria, and increased levels of antibiotic resistance among pathogens (5).

The *P*. *salmonis* type strain LF-89^T^ (ATCC VR-1361) was isolated in Chile in 1989 from a piscirickettsiosis outbreak in Coho salmon (*O*. *kisutch*) farms (6). Chilean isolates of *P*. *salmonis* have been separately clustered with the LF-89^T^ and EM-90 prototypes to form the genogroups 1 and 2, respectively (7). A hybrid group among LF-89^T^ and EM-90 prototypes has also been proposed, in addition to genogroups 3 (LF-89^T^-like isolates) and 4 (EM-90-like isolates) (7). A recent study indicated three distinct genogroups for the *P*. *salmonis* lineage on a global scale (8); that is, the NC genogroup conformed by Norwegian and Canadian isolates, and the aforementioned genogroups 1 and 2. Isolates from genogroups LF-89 and EM-90 exhibit different levels of virulence. EM-90 isolates show increased pathogenicity in post-smolt Atlantic salmon (*Salmo salar*) under *in vivo* conditions (9, 10). Furthermore, salmon infected with EM-90-like isolates can experience higher mortality rates compared to those infected with LF-89-like isolates. In fact, there are studies reporting the full survival of salmon infected with some LF-89-like isolates (11). Research involving new isolates of *P*. *salmonis* from different genogroups is required to corroborate the previously mentioned patterns. For example, *P*. *salmonis* Psal-103 and Psal-104 (which are affiliated to LF-89-like and EM-90-like prototypes, respectively) were isolated from Atlantic salmon during piscirickettsiosis outbreaks in Chilean farms. However, only a limited number of *in vitro* studies have explored the potential mechanisms through which these field isolates cause the disease. The two isolates exhibit adhesion capacity and form biofilms, with Psal-104 biofilms showing greater tolerance to salmon skin mucus (12). This finding indicates that Psal-104 cells may possess enhanced resistance to the innate immune components present in fish, such as mucus lysozyme found in rainbow trout skin mucus (13). Notably, the *in vivo* virulence of both isolates in salmonid species has yet to be assessed.

Fish affected by piscirickettsiosis exhibit clinical signs such as loss of appetite, lethargic swimming, anemia, and pale gills, as well as hemorrhagic lesions of the skin and muscle, intestinal necrosis, and pathological changes in all internal organs (1). The *in vivo* immune response of Atlantic salmon to piscirickettsiosis is well-documented (10, 14), while information on this topic in rainbow trout is less comprehensive (15). This gap in knowledge stands in contrast to the extensive research on the *in vitro* effects of *P*. *salmonis* on immune gene expression in rainbow trout cell lines (16–19). Differences in host preference (or host range) among *P*. *salmonis* genotypes could be expected considering the following points: (i) EM-90-like isolates have primarily been recovered from Atlantic salmon, (ii) LF-89-like isolates have been found indistinguishably in both Atlantic salmon and rainbow trout, and (iii) LF-89-like isolates are recovered less frequently from Coho salmon (20). Indeed, factors related to host preference and/or the *P*. *salmonis* genotype (21) may contribute to variations in fish survival to piscirickettsiosis and in the corresponding immune response.

The first study to provide evidence that *P*. *salmonis* is primarily acquired from the environment through the skin and gills of rainbow trout, rather than the oral route, was conducted by Smith et al. (22). Currently, there is limited but increasing evidence supporting the idea that the non-injured skin and gills are the main entry points for the bacterium in salmonids (23, 24). Based on this rationale, immersion infection challenge models may more accurately simulate the natural entry route of waterborne pathogens, such as *P*. *salmonis*, compared to artificially induced infections via intraperitoneal injection. Remarkably, most studies on the fish immune response to microbial infections (including piscirickettsiosis) have relied on intraperitoneal injections. Other studies have used fish cohabitation as a model for the transmission of piscirickettsiosis (10, 14), but this approach overlooks the issue of “specimen zero” in disease outbreaks, as the Trojan fish are also obtained through intraperitoneal injections. It is important to emphasize that emulating more realistic infection settings may be a key issue when evaluating the efficacy of vaccines against piscirickettsiosis.

This study aimed to determine and compare the expression of nine genes involved in the immune response of rainbow trout in the first stage of seawater farming upon *P*. *salmonis* infections. The examined genes encode three immune marker groups: (1) cell surface markers and intracellular receptors linked to cell-mediated immunity, (2) components, and (3) modulators of the adaptive and innate immune system. The former group includes genes encoding the cluster of differentiation 4 (CD4-dressed cells within T cell populations that recognize pathogens and secrete cytokines), the major histocompatibility complex class I (MHC I-dressed cells in lymphoid and epithelial tissues present antigens to T cells and natural killer cells) and MHC II (primarily associated with antigen presentation to T cells), as well as the toll-like receptor 3 (TLR-3), which is intracellularly expressed in various tissues (e.g., the liver, pyloric ceca, intestine, spleen, and head and trunk kidney). The second group involves genes encoding the primary systemic antibody immunoglobulin M (IgM) (adaptive component) and the acute-phase reactant serum amyloid A (SAA) (innate component). The third group is represented by genes encoding adaptive/innate modulators, such as pro-inflammatory cytokines (i.e., interleukin-1β [IL-1β] and tumor necrosis factor-alpha [TNF-α]) and the anti-inflammatory interleukin-10 (IL-10). In this study, *P*. *salmonis* infections were induced by seawater baths containing the type strain LF-89^T^ (genogroup LF-89) and two field isolates, Psal-103 (genogroup LF-89-like) and Psal-104 (genogroup EM-90-like).

## Materials and methods

### Sources of *P*. *salmonis*

Employed in this study were the type strain of *P*. *salmonis*, LF-89^T^ (genogroup 1), as well as two Chilean bacterial isolates, Psal-103 and Psal-104 (representative of the LF-89-like and EM-90-like prototypes; genogroups 3 and 4, respectively). These field strains were obtained from kidney samples of farmed Atlantic salmon during piscirickettsiosis outbreaks in the Los Lagos Region (Chile). Psal-103 and Psal-104 were included in the present study as both isolates have fully sequenced genomes, and the respective adherence properties and responsiveness to trout skin mucus (as first fish defensive line against waterborne pathogens) have been thoroughly characterized in previous studies (12, 13).

All *P*. *salmonis* were stored at −80 °C in Cryobille tubes (AES Laboratory, France) for long-term storage. All were routinely cultured in solid and liquid AUSTRAL-SRS media (25) for 5 d at 18 °C and 120 rpm. After bacterial growth, culture axenicity and species identity were confirmed by standard microscopy and PCR targeting a 91 bp fragment of the internal transcribed spacer (ITS) in the ribosomal operon (26).

### Rainbow trout immersion infection challenge with *P. salmonis*

Prior to immersion infection challenges, the bacterial concentration (cells mL^−1^) in cultures was determined on a light microscope at 1,000X magnification using the Breed's slide method and crystal violet dye (27). The number of colony forming units per milliliter (CFU mL^−1^) was determined through serial dilutions by plating on AUSTRAL-SRS agar plates.

Prior to transporting the fish from the fish farm to the experimental facility, 100 rainbow trout (average weight 100 g) were supplied and certified by an external private laboratory, which is validated and officially recognized by the National Fisheries and Aquaculture Service (SERNAPESCA). The certification confirmed that the fish were free of pathogens listed in List 1, as well as endemic pathogens, including *P. salmonis* and other harmful agents such as infectious pancreatic necrosis virus, infectious salmon anemia virus, *Flavobacterium psychrophilum, Yersinia ruckeri, Renibacterium salmoninarum*, and *Tenacibaculum* species (*T. maritimum, T. dicentrarchi*), among others. Upon arrived at CIMARQ, the fish were maintained for acclimation purposes in 600 L continuous-flow aerated seawater tanks for 2 months prior to *P*. *salmonis* challenges. Seawater was collected at CIMARQ and filtered through a 30-μm sand filter, decanted, and then filtered using cartridge filters (pore sizes: 20, 10, 5, and 1 μm), followed by UV-C irradiation (dose: 40 mJ cm^−2^). During the acclimatization period, key seawater parameters were monitored and maintained as follows: temperature 14.9 ± 0.6 °C, salinity 31.34 ± 1.33 ‰, and dissolved oxygen 8.0 ± 0.7 mg O2 L^−1^. Fish under acclimatization were fed with commercial food pellets at 1.5% body weight daily, subjected to a photoperiod of 12:12 light: dark, and feces were removed every other day.

After acclimation, rainbow trout were divided into five groups, each containing 16 fish. Three groups were separately immersed for 2 h in plastic tanks containing a mixture of 40 L seawater (treated as previously mentioned) and 4 L of a pure culture of each *P*. *salmonis* to achieve a final concentration of 1 × 10^7^ bacteria mL^−1^. Fish into this mixture were kept with constant aeration to provide a dissolved oxygen concentration of ~8.2 mg L^−1^. Another group of 16 fish was exposed for 2 h to a mixture of sterile AUSTRAL-SRS broth (without *P*. *salmonis*) and seawater, in the same proportion and under the same aeration conditions as used in the immersion infection challenges. This treatment is hereinafter referred to as “the sterile medium treatment” and was included to assess any influence of fish handling during the immersion infection challenges on trout gene expression patterns. In addition, a negative control group of 16 fish was used for comparison purposes.

Following exposure, fish from each group were transferred to a recirculating aquaculture system (RAS) facility and distributed among 100 L plastic tanks (work volume 80 L) at a stocking density of approximately 12 kg m^−3^, that is, 8 fish per tank. Therefore, the study was conducted in duplicate tanks. Fish were kept with daily feeding at 1.5% body weight, a photoperiod of 12:12 light: dark, and feces were removed every other day. Dead fish were removed as needed. The RAS was maintained for 30 d at 14.5 ± 1.5 °C, with a salinity of 30–32 ‰, and constant aeration to provide a dissolved oxygen concentration of 7.5 ± 0.6 mg L^−1^. Mortalities were sampled by AUSTRAL-SRS agar streaking of skin lesions and internal organs (i.e., spleen, liver, head kidney, and gills). The resulting isolates were analyzed via conventional PCR using a primer pair targeting *P*. *salmonis* (Table 1) (26).

**Table 1:** Characteristics of the primer sets used to amplify interest genes from cDNA templates.

**Primer name**	**Description**	**Target gene**	**Sequence (5′to 3′)**	**Target species**	**Amplicon size (bp)**	**Melting temp. (°C)**	**Efficiency (%)**	**References**
CD4_F	Cluster of differentiation 4	*cd4-1*	CATTAGCCTGGGTGGTCAAT	*O. mykiss*	89	55	88.2	(29)
CD4_R			CCCTTTCTTTGACAGGGAGA					
MHC I_F	Major histocompatibility complex class Ib	*Onmy-IB Mhc* region: *onmy-UCA*	TCCCTCCCTCAGTGTCT	*O. mykiss*	73	55	98.7	(30)
MHC I_R			GGGTAGAAACCTGTAGCGTG					
MHC II_F	Major histocompatibility complex class II beta-chain	*oncmyk-dbb*	CCACCTGGAGTACACACCC	*O. mykiss*	117	60	82.6	(31)
MHC II_R			TTCCTCTCAGCCTCAGGCAG					
IgM_F	Immunoglobulin M heavy chain	IGHM	CTTGGCTTGTTGACGATGAG	*O. mykiss*	72	54	80.7	(29)
IgM_R			GGCTAGTGGTGTTGAATTGG					
IL1β_F	Interleukin-1 beta 1	*il1b1*	ACATTGCCAACCTCATCATCG	*O. mykiss*	91	59	80.3	(32)
IL1β_R			TTGAGCAGGTCCTTGTCCTTG					
IL-10_F	Interleukin 10	*il10*	CGACTTTAAATCTCCCATCGAC	*O. mykiss*	70	54	83.4	(29)
IL-10_R			GCATTGGACGATCTCTTTCTTC					
TLR-3_F	Toll-like receptor 3	*tlr3*	CTTCAACAGCCTCACCA	*O. mykiss*	82	56	80.9	(31)
TLR-3_R			CTCGTTGTGCTGTACGGT					
TNF-α_F	Tumor necrosis factor alpha	*tnf-α*	TCTTACCGCTGACACAGTGC	*O. mykiss*	130	57	84.6	(33)
TNF-α_R			AGAAGCCTGGCTGTAAACGA					
SAA_F	Serum amyloid A	*saa*	GGAGATGATTCAGGGTTCCA	*O. mykiss*	78	55	94.3	(33)
SAA_R			TTACGTCCCCAGTGGTTAGC					
RTS1_F^(*)^	Internal transcribed spacer in the ribosomal operon	ITS	TGATTTTATTGTTTAGTGAGAATGA	*P. salmonis*	91	50	97.2	(26)
RTS2_R^(*)^			AAATAACCCTAAATTAATCAAGGA					

Forward (F) and reverse (R) primer names, target genes, primer sequences, target organism, size of the PCR products, melting temperatures (Tm) for annealing, amplification efficiency, and references. ^(*)^ This primer set was also used for conventional PCR from DNA templates.

Randomly selected rainbow trout were collected from duplicate RAS tanks for each *P*. *salmonis* infection group (i.e., Psal-103, Psal-104, and LF-89^T^) at 15 and 30 days post-infection (dpi). Fish collection was performed in triplicate, ensuring balanced sampling across the two RAS tanks per group. Specifically, at 15 dpi, two fish were collected from tank 1 and one fish from tank 2 for each infection group, whereas at 30 dpi, one fish was collected from tank 1 and two fish from tank 2 for the same infection group. Various samples were obtained from these fish (see details below) to compare with samples collected from fish in the sterile AUSTRAL-SRS medium treatment and from the pre-challenge condition. For the sterile medium treatment, three rainbow trout, two fish from tank 1 and one from tank 2, were collected 2 h after exposure to the treatment. The pre-challenge condition was established by collecting three rainbow trout directly from 600 L continuous-flow acclimation tanks approximately 2 h prior to the start of the immersion infection challenges.

Collected fish were euthanized using benzocaine at a concentration of 30 mg L^−1^. Subsequently, skin swabbing was performed using GenoTube swabs (Applied Biosystems) along with tissue sampling from various organs (i.e., gills, spleen, liver, muscle, and head kidney). Skin swabbing was performed by rolling a swab along the lateral line of the fish, from the operculum to the tail, following a previously described methodology (28). Swab samples were stored at −80 °C until total RNA extractions for further analysis. Tissue samples were preserved in RNAlater^TM^ solution (Ambion, Austin, TX, USA) and stored at −20 °C until total RNA extraction for RT-qPCR assays.

Additionally, triplicate 60 mL seawater samples were collected from the RAS tanks at 15 and 30 dpi, as well as from acclimation tanks at days 0 and 30 of the challenge trials. These samples were filtered using sterilized syringes with sterile 25 mm Swinnex filter holders fitted with PVDF filters (0.22 μm pore size, 25 mm diameter; GVWP02500, Millipore). The PVDF filters were air-dried, placed into sterile cryovials containing 300 μL of RNAlater^TM^ solution, and stored at −20 °C until further analysis.

### Total RNA extraction and synthesis of complementary DNA (cDNA)

Tissue samples were processed for total RNA extraction using the TRIzol™ Isolation kit (Thermo Fisher Scientific, NY, USA) with an initial tissue disruption step performed using a TissueLyser II (Qiagen, Hilden, Germany). Briefly, 20 mg of tissue were transferred to cryotubes containing a 1:1 mixture of 1.0 and 2.3 mm diameter zirconium beads (Low Binding Zirconium Beads, OPS Diagnostics, Lebanon, NJ) and 1 mL of TRIzol™. The TissueLyser II was operated for 2 × 1.5 min at a frequency of 30 Hz, with a 1 min pause between runs. Following cell disruption, the homogenate was transferred to fresh Eppendorf tubes, and total RNA was extracted according to the manufacturer's protocol for the TRIzol™ reagent. RNA extraction from skin swab samples followed the same mechanical disruption procedure, but exclusively used 200 μm diameter zirconium beads (OPS Diagnostics). Subsequently, microbial debris was transferred to fresh Eppendorf tubes for RNA extraction using the Ambion^®^ RNA extraction kit (AM1560, Ambion, Austin, TX, USA), following the manufacturer's instructions. For total RNA extraction from filtered water samples, the filters were gradually thawed on ice and processed using the same Ambion^®^ kit, incorporating a mechanical disruption step with 200 μm diameter zirconium beads and the TissueLyser II, set for 2 × 1 min at a frequency of 27 Hz. All total RNA preparations were resuspended in DEPC-treated Milli-Q water. The concentration and integrity of all RNA extracts were assessed using a Qubit 4 fluorometer (Thermo Fisher Scientific, MA, USA), employing the Qubit™ RNA HS or BR assays and IQ assay kit, respectively (Thermo Fisher Scientific). The average integrity and quality (IQ) value of the total RNA extracts was 8.7 ± 0.5. These samples were stored at −20 °C until further analysis.

To avoid DNA contamination, total RNA samples were treated with the TURBO DNA Free™ kit (Applied 500 Biosystems, Austin, TX, USA), following the manufacturer's instructions. Subsequently, cDNA was synthesized from DNA-free RNA using the ImProm-II™ Reverse Transcription System (Promega, WI, USA), according to the manufacturer's protocol. Reverse transcription reactions were performed as per the manufacturer's guidelines by using 20 ng of DNase-treated RNA and either the Oligo(dT)_15_ primer set (for host gene expression analysis) or random primer set (for *P*. *salmonis* load detection), which are commonly used for cDNA synthesis from mRNAs with and without poly(A) tails, respectively. The resulting cDNAs products were quantified using the Qubit™ ssDNA assay kit (Thermo Fisher Scientific) and stored at −20 °C for RT-qPCR analysis.

### Two-step RT-qPCR assays

The expression of nine immune-gene markers (Table 1) was quantified by qPCR from cDNA templates derived from RNA extracted from tissue samples, using specific primer sets (29–33). Similarly, transcript levels of a fragment of the ITS region in the ribosomal operon of *P*. *salmonis* (Table 1) were quantified from cDNA synthesized from rainbow trout skin swab samples. Primer sets were initially selected from the current literature (Table 1). The specificity of the chosen primers and expected amplicon sizes were evaluated using *in silico* tools available through NCBI's Primer BLAST. Only primer sets that passed this preliminary *in silico* analysis were synthesized by Integrated DNA Technologies (IDT). Before performing RT-qPCR assays, we performed *in vitro* validation of the primers via conventional PCR amplification followed by agarose gel electrophoresis. This allowed us to optimize melting temperatures and confirm the size and specificity of the amplicons.

Conventional PCR reactions were performed in a total volume of 25 μL containing 100 ng of cDNA template and the GoTaq^®^ Green Master Mix (2X, Promega, Madison, WI, USA). The thermal cycling program consisted of an initial denaturation at 94 °C for 2 min, followed by 30 cycles of: denaturation at 94 °C for 30s, annealing at the temperature specific for each primer pair (Table 1) for 30s, and extension at 72 °C for 30s. The program concluded with a final extension at 72 °C for 5 min. The GeneRuler Low Range DNA ladder (Thermo Fisher Scientific) was included in every agarose gel electrophoresis (2.5% w/v). Subsequently, selected amplicons generated by conventional PCR were excised from 2.5% (w/v) agarose gels using the Wizard™ SV genomic DNA purification kit (Promega), following the manufacturer's instructions. The concentration of each purified PCR product was measured using the Qubit™ dsDNA HS (Thermo Fisher Scientific). These purified PCR amplicons were used as templates to generate standard curves for qPCR quantification, following a method previously applied in *P*. *salmonis* studies (34). To calculate amplicon copy number, the concentration (ng μL^−1^) was divided by the amplicon's molecular mass, estimated using the formula: mass = [(n) × (M/NA)] × 10^9^ where ‘n' is the amplicon length (bp), M is the average molecular weight of a base pair (660 g mol^−1^), NA is the Avogadro's number (6,0221 × 10^23^ bp mol^−1^), and 10^9^ is the conversion factor from grams to nanograms. A six-point standard curve, starting from 4 × 10^7^ copies and prepared through serial 10-fold dilutions, was included in each qPCR run. The qPCR efficiencies and correlation coefficients (R^2^) for immunity-related genes were 85.9 ± 6.1% and 0.96 ± 0.08 (mean ± SD), respectively. For the *P*. *salmonis* ITS fragment, efficiencies were 97.2 ± 0.56%, with an R^2^ of 1.0 ± 0.0 (Table 1). RT-qPCR cycle threshold (Ct) values for target genes are depicted in Supplementary Figure 1.

The qPCR thermal cycling protocol began with an initial denaturation at 95 °C for 3 min, followed by 40 amplification cycles. Each cycle consisted of a denaturation at 95 °C for 30 s, annealing for 45 s at a temperature specific to the primer set used (Table 1), and extension at 72 °C for 45 s. All reactions were performed using a Stratagene Mx3000P real-time PCR system (Agilent Technologies-Stratagene). The specificity of the resulting PCR products was confirmed by agarose gel electrophoresis and melting curve analysis, which was conducted using MXPro software (version 4.10; Agilent Technologies).

### Statistical analysis

The number of gene-transcript copies was calibrated by the total RNA concentration per sample to addresses potential troubles raised during RNA preparation (e.g., RNA quantity and integrity). Calibrated gene-transcript copies were used to calculate the log_2_-fold change (log_2_-FC) in gene expression between rainbow trout from infectious treatments (with different *P*. *salmonis*) at 15 and 30 dpi and those collected from the pre-challenge condition, or between *P*. *salmonis*-challenged trout at 15 and 30 dpi and those collected from the sterile medium treatment. Differential gene expression analysis was performed using the “DESeq2” package and the DESeq function (35). Immune gene markers with statistically significant log_2_-FC values (i.e., adjusted P-values [*P*-adj] ≤ 0.05), and thresholds of ≥2 and ≤ −2 were considered up- and downregulated, respectively. Log_2_-FC data and respective statistical significance were visualized using the Pheatmap package (v1.0. 12) in R (36). Additionally, cumulative mortality curves of rainbow trout were generated from data obtained from seawater immersion infection challenges using the GraphPad Prism 10.4.1 software (GraphPad Software, Inc., CA, USA). Gehan-Breslow-Wilcoxon and Log-rank (Mantel-Cox) tests were used to assess the statistically significant differences between resulting curves (*P* ≤ 0.05).

## Results

### Detection of *P. salmonis* during early piscirickettsiosis and rainbow trout survival

**Figure 1:**
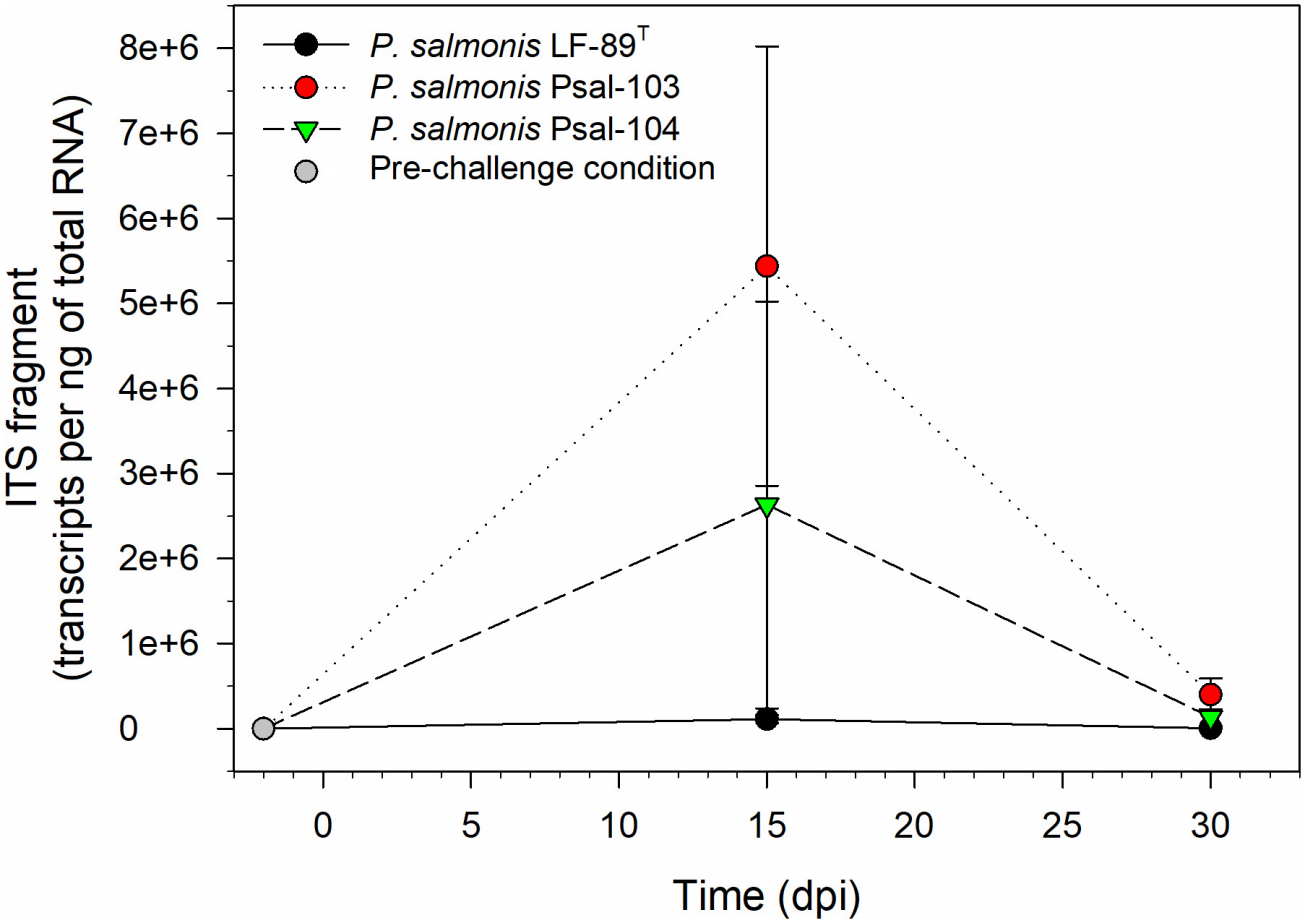
*Piscirickettsia salmonis* ITS fragment transcripts in skin swab samples from randomly collected surviving rainbow trout at different time points. The gray circle denotes the load of skin-associated *P*. *salmonis* in the pre-challenge condition, which showed undetectable. Black, red, and green symbols indicate the skin-associated bacterial load for the three *P*. *salmonis* at 15 and 30 dpi. Symbols depict mean values ± standard error (*n* = 3).

ITS transcripts of *P*. *salmonis* were undetectable in skin swab samples from rainbow trout in the pre-challenge condition (Figure 1). These transcripts were also undetectable in seawater samples collected from acclimation tanks at days 0 and 30 of the challenge trials, as well as in skin samples from rainbow trout in the sterile medium treatment, which were collected 2 h after exposure to the treatment. The number of ITS transcripts in RAS seawater samples was 2.21 × 10^4^ ± 8.99 × 10^3^ gene copies per ng of total RNA at 15 dpi, but transcripts were undetectable at 30 dpi. *P*. *salmonis* transcripts were also undetectable in non-skin tissues of randomly selected surviving rainbow trout after the bath challenges, except in some mortalities from the infectious treatments, as detected by conventional PCR at 30 dpi (Supplementary Figure 2). Maximum loads of skin-associated *P*. *salmonis* in surviving rainbow trout were detected at 15 dpi, but this remarkably decreased at 30 dpi, especially for *P*. *salmonis* Psal-103- and Psal-104-challenged trout (Figure 1). At both 15 and 30 dpi, the *P*. *salmonis* load (measured as ITS transcripts of the bacterium) in rainbow trout skin, in decreasing order of abundance, was as follows: Psal-103 > Psal-104 > LF-89^T^. For instance, at 15 dpi, the bacterial load of *P*. *salmonis* was 5.44 ± 2.58 × 10^6^ and 1.12 × 10^5^ ± 4.71 × 10^4^ copies per ng of total RNA in the skin of Psal-103- and LF-89^T^-infected trout, respectively (Figure 1). For comparison purposes, at 30 dpi, the load of *P*. *salmonis* ITS transcripts in the skin of Psal-104-caused mortalities reached 7.45 × 10^7^ ± 4.0 × 10^6^ copies per ng of total RNA.

**Figure 2:**
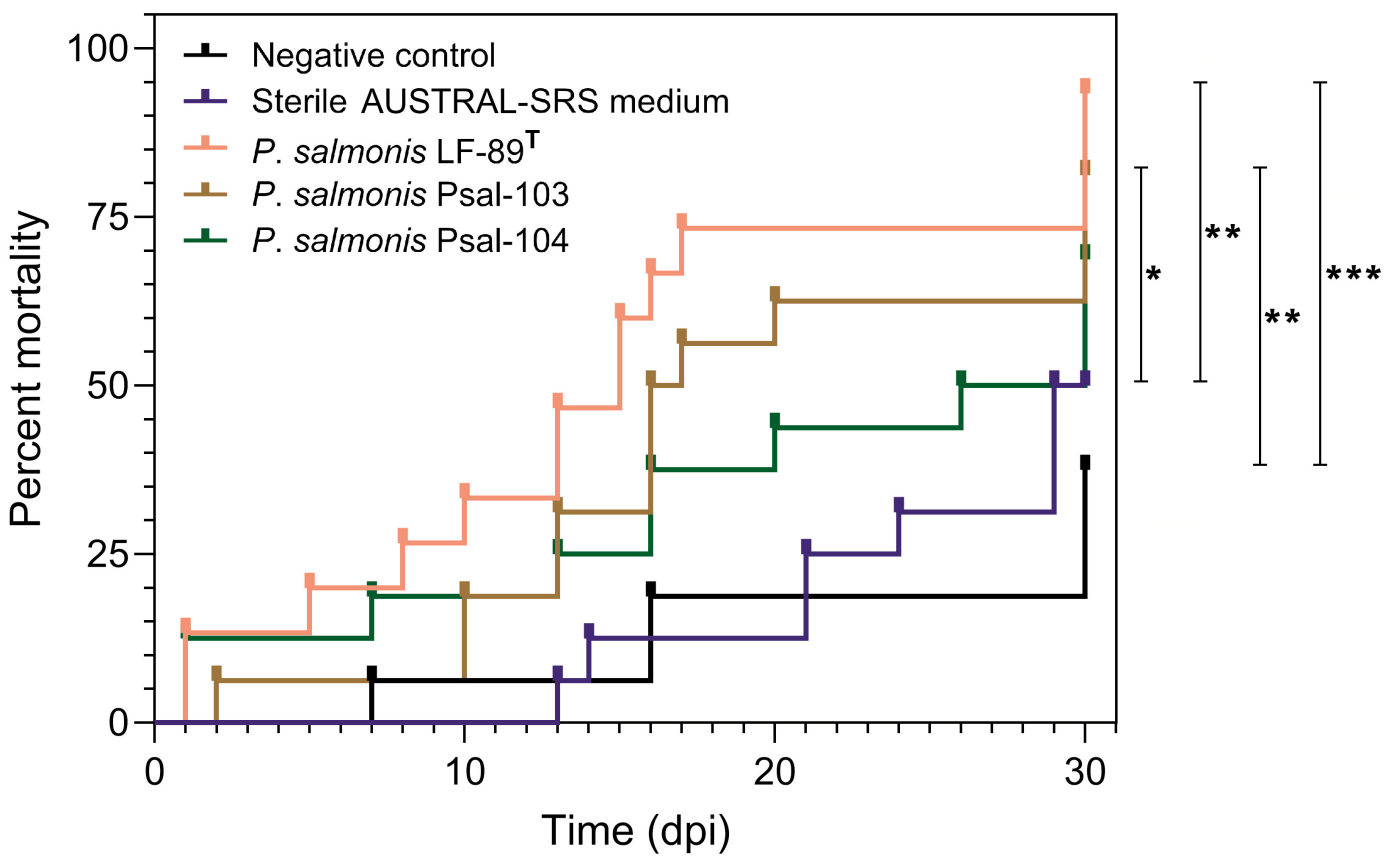
Probability of rainbow trout mortality over time. Rainbow trout mortality curves from the negative control group (black line), the sterile SRS medium treatment (blue line), and infectious treatments with *P*. *salmonis* of three different genotypes (pink, brown, and green lines). Only comparisons between two curves with statistically significant differences using the Gehan-Breslow-Wilcoxon and Log-rank test (*p* ≤ 0.05 = *, *p* ≤ 0.01 = **, *p* ≤ 0.001 = ***) are pointed out. Deaths at the first day in two *P*. *salmonis* infectious treatments were caused by escape mortality.

There were no significant differences in mortality between rainbow trout from the negative control group and those from the sterile medium treatment (Figure 2). Similarly, no significant differences were observed among the infectious treatment groups (Figure 2). The percent mortality in the negative control group was significantly lower (*P* ≤ 0.05, Gehan-Breslow-Wilcoxon and Log-rank tests) than in the LF-89^T^- and Psal-103-infected groups (Figure 2). Fish from the sterile AUSTRAL-SRS medium treatment also showed a significantly lower cumulative mortality (*P* ≤ 0.05, Gehan-Breslow-Wilcoxon and Log-rank tests) compared to the LF-89^T^- and Psal-103-infected rainbow trout (Figure 2). Although not statistically significant, cumulative mortality in the negative control and sterile medium groups tended to be lower than in the Psal-104-infected group (Figure 2).

### Differential expression of immune gene markers

**Figure 3:**
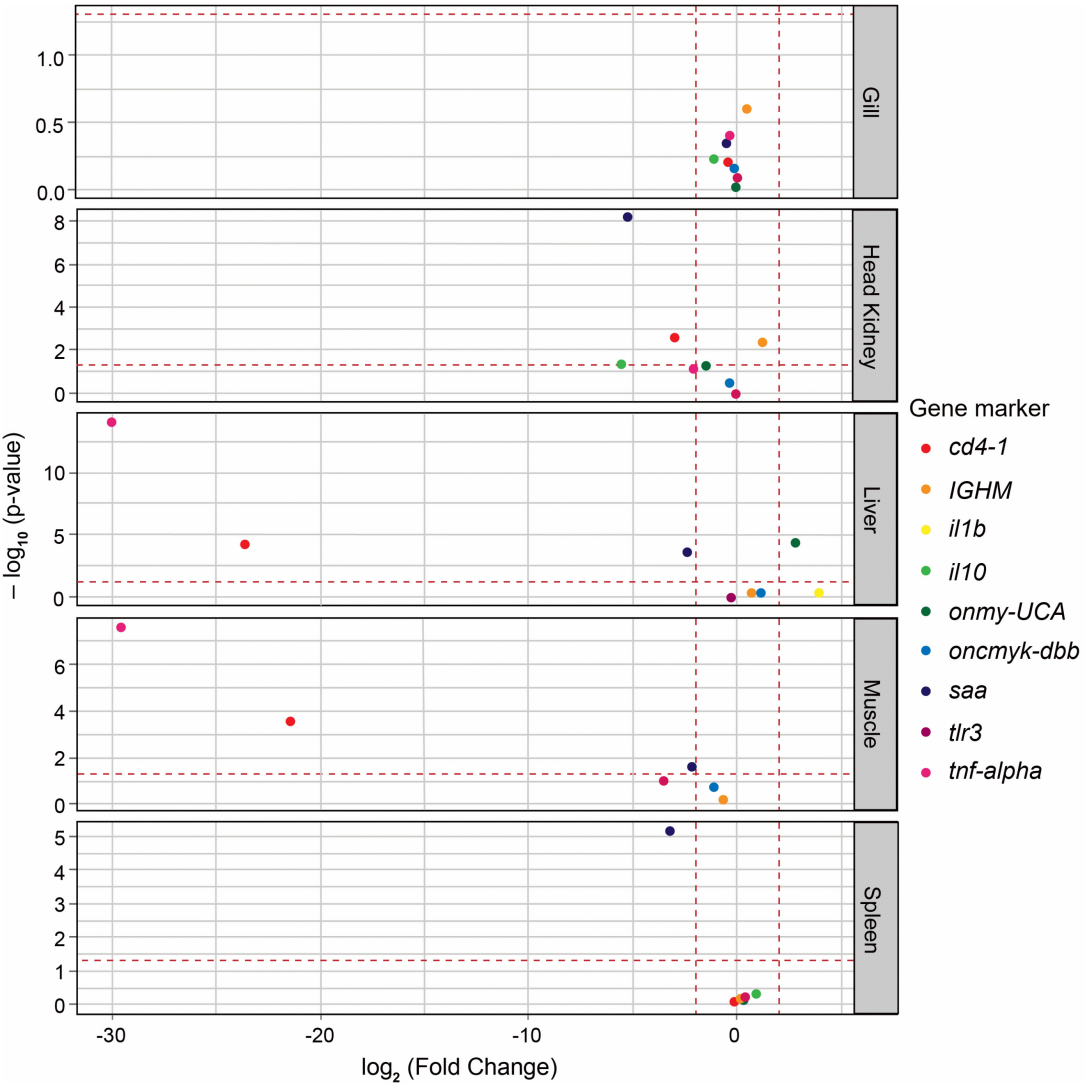
Volcano plot displaying the changes in immune gene expression (log_2_-FC) between the sterile medium treatment and the pre-challenge condition. The fold change (FC) values for all genes are plotted on the X-axis, while the negative logarithmic adjusted *p*-values (Padj) are shown on the Y-axis. The horizontal dashed line denotes the cutoff value of 0.05 for the negative logarithmic Padj-value. The two vertical dashed lines denote the cutoff value of −2 and 2 for the log_2_-FC. Each immune gene is represented by a differently colored circle. Genes located within the range of −2 to 2 and with Padj values of ≤ 0.05 were considered to show no significant variation between the compared conditions.

**Figure 4:**
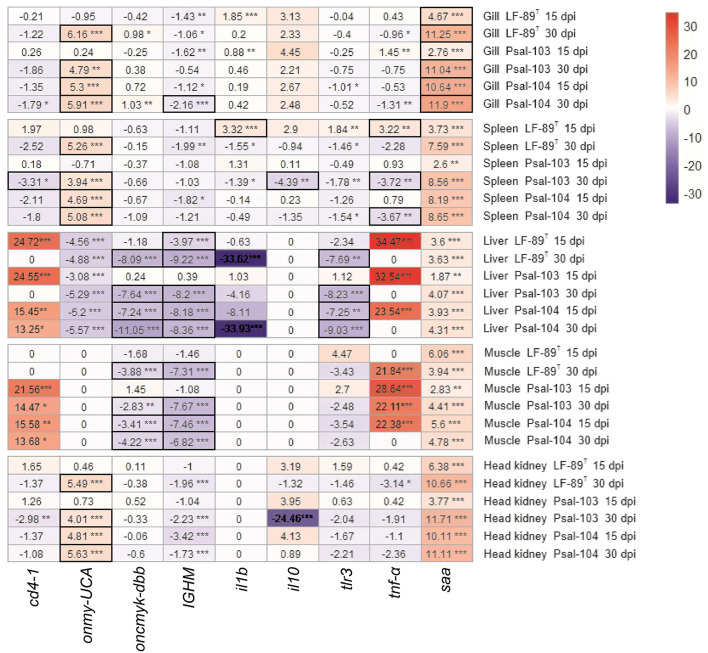
Heat map of fold change in gene expression between immersion-infected rainbow trout and those in a pre-challenge condition. Nine immune gene markers were analyzed in various tissues of rainbow trout infected with *P*. *salmonis* (LF-89^T^, Psal-103, and Psal-104) at 15 and 30 dpi. The resulting level expressions were compared to those determined for the same immune gene markers in the tissues of rainbow trout under the pre-challenge condition. An immune gene marker was considered significantly up- or downregulated (Padj-values ≤ 0.05) if it had expression levels with log_2_-fold changes (log_2_-FC) cutoffs of ≥ 2 (red scale) or ≤ −2 (blue scale), respectively. These cutoffs allow for the identification of genes with substantial changes in their expression levels. FC values that were not significant or fell outside these defined thresholds were not considered differential gene expression. The significance levels are indicated by asterisks: *p ≤ 0.05, **p ≤ 0.01, ***p ≤ 0.001. Heat map cells with thick outlines display genes responsive to *P*. *salmonis* (see explanation in the main text).

Log_2_-FC values were calculated to identify significant variations in the expression of immune marker genes between the sterile medium treatment and the pre-challenge condition. The null hypothesis asserts that, in the absence of any effects from fish handling, no significant differences in gene expression between these two conditions are expected. Therefore, genes with log_2_-FC values between ≤ 2 and ≥−2, with at a P-adj value of ≤ 0.05, were considered to have not changed significantly. These cutoffs were met for some of the tested genes, depending on the tissue sample. Specifically, this was true for the gills (all genes), head kidney (*onmy-UCA, oncmyk-dbb*, and *tlr3*), liver (*IGHM, oncmyk-dbb*, and *tlr3*), muscle (*IGHM* and *oncmyk-dbb*), and spleen (all genes except *saa*) (Figure 3). In addition, among the genes with log_2_-FC that fell within these threshold values (i.e., ≤ 2 and ≥−2), only those with significant log_2_-FC values of ≥ 2 or ≤ −2, when comparing infectious treatments to the pre-challenge condition, were associated with the differential expression caused by *P*. *salmonis* (see heat map cells with thick outlines in Figure 4).

Immune markers that showed significant levels of gene regulation induced by *P*. *salmonis* included *onmy-UCA* and *saa*. These genes were significantly upregulated in the gills of surviving trout, and *onmy-UCA* was also upregulated in the spleen and head kidney (Figure 4). By contrast, the genes *oncmyk-dbb* and *IGHM* were significantly downregulated in the liver and muscle, along with *tlr3* in the liver (Figure 4). In addition, the expression of *tnf-*α varied in the spleen depending on the strain and the post-infection time. This gene was significantly upregulated at 15 dpi in the spleen of LF-89^T^-infected surviving rainbow trout; however, in trout infected with Psal-103 and Psal-104, *tnf-*α was significantly downregulated (Figure 4). Additionally, there were interesting findings regarding gene upregulation that could not be exclusively attributed to *P*. *salmonis*. This was observed in the spleen, liver, muscle, and head kidney for *saa*, as well as for *cd4-1* and *tnf-*α in the liver and muscle of surviving trout (Figure 4).

## Discussion

RT-qPCR analysis and other cDNA-dependent approaches are often used for studying the active fraction of predominant bacterial populations by detecting sequence transcripts (37–39). In the present study, *P*. *salmonis* was detected by RT-qPCR in seawater samples collected from the pilot-scale RAS at 14.5 ± 1.5 °C at 15 dpi, but not at 30 dpi. A prior study used ITS DNA-based qPCR to analyze the water column of aquaculture settings in southern Chile (40); this study detected *P*. *salmonis* up to 30 days (but not more than 40 to 50 days) after cage emptying. In addition, *P*. *salmonis* has been reported to survive extracellularly in seawater at 15 °C for approximately 12 days (41). Therefore, although *P*. *salmonis* can persist in aquaculture settings for at least 30 days after the removal of affected fish, the presently obtained results indicate that only shorter periods ensure the detection of its potential activity by RT-qPCR outside hosts. These results suggest that a potential interference of free-living *P*. *salmonis* in the pilot-scale RAS was unlikely or minimal.

*Piscirickettsia salmonis* was detected in skin samples from randomly collected rainbow trout at 15 and 30 dpi (Figure 1), but not in non-skin tissues from these fish, except in some samples randomly collected from mortalities at 30 dpi, and that were analyzed by conventional PCR (Supplementary Figure 2). These observations indicate that the applied sampling strategy, focused on the analysis of samples taken from surviving fish, allowed the collection of data in the first stages of piscirickettsiosis in rainbow trout. The load of skin-associated *P*. *salmonis* in rainbow trout varied from undetectable in the pre-challenge condition to detectable levels at 30 dpi, with a peak at 15 dpi (Figure 1). In the absence of data on the load of *P*. *salmonis* in rainbow trout challenged with the bacterium, studies conducted in other salmonid species can be used for comparison purposes. For instance, the load of rainbow trout skin-associated *P*. *salmonis* in the present study was in the range reported (between 30 and 65 dpi) in the muscle of Atlantic salmon, but compared with head kidney samples (42), the load of trout skin-associated *P*. *salmonis* was higher in the current study. In addition, the load of *P*. *salmonis* in the head kidney of Atlantic salmon parrs has been determined by counting of ITS transcripts (43), with results following a bell-shaped pattern between 7 and 42 dpi, with peaks between 13 and 21 dpi. When considering the pre-challenge condition, the present study also reports that the load of trout skin-associated *P*. *salmonis* appears to follow a bell-like shape trend. However, it would be necessary to increase the sampling frequency during the initial post-challenge hours to confirm this trend. The observed decrease in skin-associated *P*. *salmonis* load toward the end of the study may reflect bacterial clearance from fish. The clearance process has been described in salmonids and represents a key and complex aspect of the disease's natural history. This process can be modulated at multiple interconnected levels, including the participation of non-specific antibodies (44) and autophagy (45). At the skin level, bacterial clearance may occur through secretion of mucus, which traps and sheds microbes. Moreover, rainbow trout skin mucus contains several antibacterial components from both the innate and adaptive immune responses (46, 47), contributing to the clearance processes.

Rainbow trout from the negative control group and sterile medium treatment exhibited the lowest mortalities (Figure 2). Over time, these fish exhibited hyperactive behaviors, contrasting with the lethargy and loss of appetite observed in infected fish. The hyperactivity was linked to aggressive social interactions, such as caudal fin biting or nipping, which could have contributed to the mortalities in the control group and sterile medium treatment. Most of the recorded mortalities started from day 7 onward (Figure 2). A prior study on *P*. *salmonis*-challenged rainbow trout by immersion baths recorded the first mortalities around day 10, leading to cumulative mortalities of 91.7% at 23 dpi (48). Rainbow trout challenged by intraperitoneal injection of *P*. *salmonis* experienced mortalities from day 4 onward, continuing up to 33 dpi (22). Mortality percentages of rainbow trout infected with *P*. *salmonis* LF-89^T^, Psal-103, and Psal-104 did not show significant differences (Figure 2). In other salmonids such as Atlantic salmon, an EM-90-like isolate provoked a higher cumulative mortality in shorter infection times compared to an LF-89-like isolate (9). Moreover, Atlantic salmon might survive challenge trials with some *P*. *salmonis* LF-89-like isolates but not with EM-90-like isolates (11). However, another study conducted in Atlantic salmon has found no differences in cumulative mortality (or time to mortality) when comparing the effects of LF-89-like and EM-90-like *P*. *salmonis* isolates (49). Our study likely required a larger sample size to detect potential differences in mortality among genotypes. However, the lack of differences in mortality between infectious treatments does not necessarily imply that gene expression responses were identical. For instance, the isolate Psal-104 (EM-90-like genotype) tended to induce higher cumulative gene regulation levels over time compared to the other two *P*. *salmonis* (Supplementary Figure 3). This pattern was observed regardless of whether the gene regulation response met the established criteria for reliable association to solely *P*. *salmonis* (Figure 3). This pattern aligns with a previous study that compared the effects of LF-89-like and EM-90-like *P*. *salmonis* isolates on gene expression in post-smolt Atlantic salmon, reporting an exacerbated immune response in PS-EM-90-infected fish, the isolate of higher pathogenicity (10). Although only a few studies have investigated the virulence mechanisms of the Psal-103 and Psal-104 isolates (12, 13), current evidence suggests that the EM-90-like isolate (Psal-104) has a greater potential capacity to overcome the trout mucosal barriers (13).

Genes encoding immune markers such as CD4, IL-1β, and IL-10 did not appear to show a direct responsiveness to *P*. *salmonis* in the tested tissues (Figure 4). The gene *tlr3* was often downregulated in various organs (particularly in the liver), or it did not show differential expression (Figure 4). This results aligns with the primary role of TLR-3 in the antiviral immunity of rainbow trout (50, 51), rather than in defending against bacterial infections (52). However, bacterial pathogens like *Aeromonas salmonicida* can also cause the downregulation of the TLR-3-encoding gene in various tissues of rainbow trout compared to controls (53). While TLR-3 is generally considered less important for recognizing bacterial ligands, with only a few examples in fish immunology literature suggesting otherwise (54), the immune response to *P*. *salmonis* deserves further investigation. For instance, the risk of secondary infections may increase due to the downregulation of TLR-3 caused by *P*. *salmonis*. This is particularly concerning in the context of emerging infectious diseases, as it could increase the susceptibility of rainbow trout to marine viruses affecting multiple organs, especially the liver.

The *oncmyk-dbb* gene, which encodes MHC-II, showed regulation levels that varied from non-differentially expressed to being downregulated in the present study (Figure 4). It was significantly downregulated in the liver and muscle of *P*. *salmonis*-infected trout, which was accompanied by a significant upregulation of *cd4-1* (a T lymphocyte gene marker) in these tissues (Figure 4). In Atlantic salmon, *P*. *salmonis* LF-89-like and EM-90-like isolates also reduced the expression of a MHC-II-encoding gene (10). The present study suggests that *P*. *salmonis* manipulates the activation of CD4+ T cells to escape the host immune response, as CD4+ T cells may be present but cannot detect antigen-presentation cues. Indeed, *P*. *salmonis* is known to evade the immune system of salmonids by downregulating MHC-II on the surface of infected cells, leading to reduced antigen presentation to CD4+ T cells and, consequently, preventing their activation (1). However, differences in *cd4-1* expression observed between the pre-challenge condition and sterile medium treatment (Figure 3) suggest that the potential proliferation of CD4+ T cells in the liver and muscle was possibly not induced by *P*. *salmonis* alone. Pathogenic infections are a major influencing factor of CD4+ T cell proliferation in fish, but they are not the only one. For example, the interaction between a fish's immune system and its microbiota can activate the innate immune response, which may promote the proliferation and activation of CD4+ T cells (55). Thus, the presence of *P*. *salmonis* along with changes in fish's commensal microbiota potentially induced by the sterile medium treatment, could have been responsible for *cd4-1* overexpression in both the liver and muscle.

The *IGHM* gene remained predominantly downregulated in all tissues, indicating that the IgM-based humoral immune response may not have been induced by *P*. *salmonis*. This response was perceived especially in the liver and muscle (Figure 4). Currently, there is limited information regarding the expression of IgM-related genes in rainbow trout infected by *P*. *salmonis*. A previous study reported reduced expression of a gene encoding IgM in the head kidney of Atlantic salmon infected by LF-89-like and EM-90-like *P*. *salmonis* isolates, particularly in fish infected with PS-EM-90 (10). This study also indicated that fish infected by PS-LF-89 showed higher variability in MHC-I gene regulation (with increased expression at 56 dpi in survivor fish), whereas PS-EM-90-infected fish showed higher expression levels from 35 dpi onwards. In the present study, the expression of the *onmy-UCA* gene, which encodes MHC-I, varied across different tissues. In the muscle and liver, *onmy-UCA* exhibited responses ranged from non-differential expression to downregulation after infections. In contrast, in the gills, spleen, and head kidney, this gene was often upregulated, which suggests an adaptive immune surveillance involving presenting antigens to CD8+ T cells to identify and eliminate *P*. *salmonis*-infected cells. Although a gene marker for CD8+ T cells was not analyzed, the increased TNF-α levels in rainbow trout's liver and muscle could stimulate T cell maturation in the thymus, potentially including CD8+ T cells (56).

*P*. *salmonis* triggers the overexpression of *il10* in the rainbow trout macrophage-like cell line (RTS11) during the early stages of infection. This overexpression helps prevent the inflammatory response and was associated with pathogen survival within macrophages (16). In the current study, however, we did not observe an anti-inflammatory response based on the overexpression of *il10* due to *P*. *salmonis*. In addition, the gene expression levels of *saa* and *tnf-*α indicated an inflammatory response, but with no involvement of the pro-inflammatory *il1b1* gene. A high-frequency sampling approach could have allowed us to detect *il1b1* expression during the initial hours post-challenge, as demonstrated by studies on *Y*. *ruckeri*-infected rainbow trout (57, 58). In the present study, the innate inflammatory response was found to be influenced not only by pathogenic stressors but also by other sources of activation. This is suggested by considering the upregulation of the *saa* gene in the spleen, liver, muscle, and head kidney, as well as the increased expression levels of the *tnf-*α gene in the liver and muscle. Indeed, the expression of the *saa* gene in rainbow trout has already been associated with both pathogenic stimuli, such as whole cells and their virulence macromolecules, and non-pathogenic stimuli, such as heavy metals and confinement (59, 60).

## Conclusions

We conducted seawater immersion bath challenges using three distinct genotypes of *P*. *salmonis* to investigate the early stages of piscirickettsiosis in rainbow trout over a 30-day period. The analysis of the surviving rainbow trout after the bath challenges indicated that genes associated with immune markers, such as CD4, IL-1β, IL-10 and TNF-α, showed minimal response (in gene regulation) to infections caused by *P*. *salmonis*. Overall, our findings suggest an innate inflammatory response in surviving fish that was related to the infection by *P*. *salmonis*, but also to non-pathogenic stimuli. The inflammatory response was associated with the expression of *saa* (e.g., in the gills) and *tnf-*α genes (in the liver and muscle), but with no involvement of the pro-inflammatory *il1b1* gene. The downregulation of *oncmyk-dbb* and *IGHM* genes indicates that *P*. *salmonis* can interfere with the activation of CD4+ T cells and impair humoral immunity in surviving fish. However, further research is needed to determine whether the response of the gene *onmy-UCA* in *P*. *salmonis*-infected rainbow trout was related to the transcriptional activation of CD8+ T cell gene markers. Notably, while there were no significant differences in mortality among the three *P*. *salmonis* infectious treatments, the isolate Psal-104 (an EM-90-like genotype) tended to induce higher cumulative levels of gene regulation over time compared to the other two genotypes. In turn. this suggests that Psal-104 may have a greater immunogenic potential. Finally, since the bacterium was found in the skin of the challenged surviving trout but not in their internal organs, our findings support the use of non-lethal and non-invasive analysis of fish skin for the early surveillance of piscirickettsiosis.

## Data Availability

The original contributions presented in the study are included in the article/Supplementary material, further inquiries can be directed to the corresponding authors.

## References

[1] Rozas-Serri M. Why does *Piscirickettsia salmonis* break the immunological paradigm in farmed salmon? biological context to understand the relative control of piscirickettsiosis. Front Immunol. (2022) 13:856896. doi: 10.3389/fimmu.2022.856896 35386699 PMC8979166

[2] Jones SR Long A MacWilliams C Polinski M Garver K. Factors associated with severity of naturally occurring piscirickettsiosis in netpen-and tank-reared juvenile Atlantic salmon at a research aquarium in western Canada. J Fish Dis. (2020) 43:49–55. doi: 10.1111/jfd.13102 31709554 PMC6972981

[3] SERNAPESCA. Informe con antecedentes sanitarios de agua dulce y mar. Departamento de salud animal subdirección de acuicultura, Servicio Nacional de Pesca y Acuicultura, septiembre. (2024). Available online at: https://www.sernapesca.cl/informes-y-datos/resultados-de-gestion/ (Accessed April 29, 2025).

[4] Enright WJ. Focus: fish health. Intrafish. (2003) 1:12–9.

[5] Avendaño-Herrera R Mancilla M Miranda CD. Use of antimicrobials in Chilean salmon farming: facts, myths and perspectives. Rev Aquac. (2023) 15:89–111. doi: 10.1111/raq.12702

[6] Fryer JL Lannan CN Giovannoni SJ Wood ND. Piscirickettsia salmonis gen. nov, sp nov, the causative agent of an epizootic disease in salmonid fishes. Int J Syst Evol Microbiol. (1992) 42:120–6. doi: 10.1099/00207713-42-1-120 1371057

[7] Isla A Saldarriaga-Córdoba M Fuentes DE Albornoz R Haussmann D Mancilla-Schulz J . Multilocus sequence typing detects new *Piscirickettsia salmonis* hybrid genogroup in Chilean fish farms: evidence for genetic diversity and population structure. J Fish Dis. (2019) 42:721–737. doi: 10.1111/jfd.12976 30851000

[8] Schober I Bunk B Carril G Freese HM Ojeda N Riedel T . Ongoing diversification of the global fish pathogen *Piscirickettsia salmonis* through genetic isolation and transposition bursts. ISME J. (2023) 17:2247–58. doi: 10.1038/s41396-023-01531-9 37853183 PMC10689435

[9] Rozas-Serri M Ildefonso R Peña A Enríquez R Barrientos S Maldonado L. Comparative pathogenesis of piscirickettsiosis in Atlantic salmon (*Salmo salar* L.) post-smolt experimentally challenged with LF-89-like and EM-90-like *Piscirickettsia salmonis* isolates. J Fish Dis. (2017) 40: 1451-1472. doi: 10.1111/jfd.12671 28745821

[10] Rozas-Serri M Peña A Arriagada G Enríquez R Maldonado L. Comparison of gene expression in post-smolt Atlantic salmon challenged by LF-89-like and EM-90-like *Piscirickettsia salmonis* isolates reveals differences in the immune response associated with pathogenicity. J Fish Dis. (2018) 41: 539-552. doi: 10.1111/jfd.12756 29143962

[11] Carril G Morales-Lange B Løvoll M Inami M Winther-Larsen HC Øverland M . Salmonid Rickettsial Septicemia (SRS) disease dynamics and Atlantic salmon immune response to *Piscirickettsia salmonis* LF-89 and EM-90 co-infection. Vet Res. (2024) 55:102. doi: 10.1186/s13567-024-01356-0 39152462 PMC11328376

[12] Levipan HA Irgang R Yáñez A Avendaño-Herrera R. Improved understanding of biofilm development by *Piscirickettsia salmonis* reveals potential risks for the persistence and dissemination of piscirickettsiosis. Sci Rep. (2020) 10:12224. doi: 10.1038/s41598-020-68990-4 32699383 PMC7376020

[13] Levipan HA Avendaño-Herrera R. Assessing the impacts of skin mucus from *Salmo salar* and *Oncorhynchus mykiss* on the growth and *in vitro* infectivity of the fish pathogen *Piscirickettsia salmonis*. *J Fish Dis*. (2021) 44: 181-190. doi: 10.1111/jfd.13275 33006764

[14] Rozas-Serri M Peña A Maldonado L. Transcriptomic profiles of post-smolt Atlantic salmon challenged with *Piscirickettsia salmonis* reveal a strategy to evade the adaptive immune response and modify cell-autonomous immunity. Dev Comp Immunol. (2018) 81:348–62. doi: 10.1016/j.dci.2017.12.023 29288676

[15] Carrizo V Valenzuela CA Aros C Dettleff P Valenzuela-Muñoz V Gallardo-Escarate C . Transcriptomic analysis reveals a *Piscirickettsia salmonis*-induced early inflammatory response in rainbow trout skeletal muscle. Comp Biochem Physiol Part D Genomics Proteomics. (2021) 39:100859. doi: 10.1016/j.cbd.2021.100859 34087760

[16] Álvarez CA Gomez FA Mercado L Ramírez R Marshall SH. Piscirickettsia salmonis imbalances the innate immune response to succeed in a productive infection in a salmonid cell line model. PLoS ONE. (2016) 11:e0163943. doi: 10.1371/journal.pone.0163943 PMC5056700 27723816

[17] Peña B Isla A Haussmann D Figueroa J. Immunostimulatory effect of salmon prolactin on expression of Toll-like receptors in *Oncorhynchus mykiss* infected with *Piscirickettsia salmonis*. *Fish Physiol Biochem*. (2016) 42:509–16. doi: 10.1007/s10695-015-0155-5 26537800

[18] Morales-Lange B Nombela I Ortega-Villaizán MDM Imarai M Schmitt P Mercado L. Induction of *foxp3* during the crosstalk between antigen presenting like-cells MHCII^+^CD83^+^ and splenocytes CD4^+^IgM^−^ in rainbow trout. Biology. (2021) 10:324. doi: 10.3390/biology10040324 33924548 PMC8069158

[19] Valenzuela CA Azúa M Álvarez CA Schmitt P Ojeda N Mercado L. Evidence of the autophagic process during the fish immune response of skeletal muscle cells against *Piscirickettsia salmonis*. *Animals*. (2023) 13:880. doi: 10.3390/ani13050880 36899738 PMC10000225

[20] Saavedra J Hernandez N Osses A Castillo A Cancino A Grothusen H . Prevalence, geographic distribution and phenotypic differences of *Piscirickettsia salmonis* EM-90-like isolates. J Fish Dis. (2017) 40: 1055-1063. doi: 10.1111/jfd.12581 28075013

[21] Nourdin-Galindo G Sánchez P Molina CF Espinoza-Rojas DA Oliver C Ruiz P . Comparative pan-genome analysis of *Piscirickettsia salmonis* reveals genomic divergences within genogroups. Front Cell Infect Microbiol. (2017) 7:459. doi: 10.3389/fcimb.2017.00459 29164068 PMC5671498

[22] Smith PA Pizarro P Ojeda P Contreras J Oyanedel S Larenas J. Routes of entry of *Piscirickettsia salmonis* in rainbow trout *Oncorhynchus mykiss*. *Dis Aquat Organ*. (1999) 37:165–72. doi: 10.3354/dao037165 10546046

[23] Long A Goodall A Jones SR. Development of a *Piscirickettsia salmonis* immersion challenge model to investigate the comparative susceptibility of three salmon species. J Fish Dis. (2021) 44: 1-9. doi: 10.1111/jfd.13261 33067883 PMC7756497

[24] Coca Y Godoy M Pontigo JP Caro D Maracaja-Coutinho V Arias-Carrasco R . Bacterial networks in Atlantic salmon with piscirickettsiosis. Sci Rep. (2023) 13:17321. doi: 10.1038/s41598-023-43345-x 37833268 PMC10576039

[25] Yañez AJ Valenzuela K Silva H Retamales J Romero A Enriquez R . Broth medium for the successful culture of the fish pathogen *Piscirickettsia salmonis*. *Dis Aquat Org*. (2012) 97:197–205. doi: 10.3354/dao02403 22422090

[26] Marshall S Heath S Henríquez V Orrego C. Minimally invasive detection of *Piscirickettsia salmonis* in cultivated salmonids via the PCR. Appl Environ Microbiol. (1998) 64:3066–9. doi: 10.1128/AEM.64.8.3066-3069.1998 9687475 PMC106817

[27] Hanks JH James DF. The enumeration of bacteria by the microscopic method. J Bacteriol. (1940) 39:297–305. doi: 10.1128/jb.39.3.297-305.1940 16560293 PMC374573

[28] Avendaño-Herrera R Tralma L Wicki H Barrios-Henríquez F Levipan HA. Skin-mucus prokaryote community of Atlantic salmon (*Salmo salar*) in response to bath challenge with *Tenacibaculum dicentrarchi*. *J Fish Dis*. (2025) 2025:e14157. doi: 10.1111/jfd.14157 40495401

[29] Raida MK Buchmann K. Development of adaptive immunity in rainbow trout, *Oncorhynchus mykiss* (Walbaum) surviving an infection with *Yersinia ruckeri*. *Fish Shellfish Immunol*. (2008) 25:533–41. doi: 10.1016/j.fsi.2008.07.008 18706505

[30] Skov J Kania PW Holten-Andersen L Fouz B Buchmann K. Immunomodulatory effects of dietary β-1, 3-glucan from *Euglena gracilis* in rainbow trout (*Oncorhynchus mykiss*) immersion vaccinated against *Yersinia ruckeri*. *Fish Shellfish Immunol*. (2012) 33:111–20. doi: 10.1016/j.fsi.2012.04.009 22548789

[31] Christie L van Aerle R Paley RK Verner-Jeffreys DW Tidbury H Green M . The skin immune response of rainbow trout, *Oncorhynchus mykiss* (Walbaum), associated with puffy skin disease (PSD). Fish Shellfish Immunol. (2018) 78:355–63. doi: 10.1016/j.fsi.2018.04.053 29709592

[32] Musharrafieh R Tacchi L Trujeque J LaPatra S Salinas I. Staphylococcus warneri, a resident skin commensal of rainbow trout (*Oncorhynchus mykiss*) with pathobiont characteristics. Vet Microbiol. (2014) 169:80–8. doi: 10.1016/j.vetmic.2013.12.012 PMC3967797 24438987

[33] Evenhuis JP Cleveland BM. Modulation of rainbow trout (*Oncorhynchus mykiss*) intestinal immune gene expression following bacterial challenge. Vet Immunol Immunopathol. (2012) 146:8–17. doi: 10.1016/j.vetimm.2012.01.008 22341800

[34] Zúñiga A Aravena P Pulgar R Travisany D Ortiz-Severín J Chávez FP . Transcriptomic changes of *piscirickettsia salmonis* during intracellular growth in a salmon macrophage-like cell line. Front Cell Infect Microbiol. (2020) 9:426. doi: 10.3389/fcimb.2019.00426 31998656 PMC6964531

[35] Love MI Huber W Anders S. Moderated estimation of fold change and dispersion for RNA-seq data with DESeq2. Genome Biol. (2014) 15:550. doi: 10.1186/s13059-014-0550-8 25516281 PMC4302049

[36] Kolde R. pheatmap: Pretty Heatmaps. R Package Version 1.0.12. (2019). Available online at: https://cran.r-project.org/web/packages/pheatmap/pheatmap.pdf (Accessed April 15, 2025).

[37] Stoeva MK Aris-Brosou S Chételat J Hintelmann H Pelletier P Poulain AJ. Microbial community structure in lake and wetland sediments from a high Arctic polar desert revealed by targeted transcriptomics. PLoS ONE. (2014) 9:e89531. doi: 10.1371/journal.pone.0089531 24594936 PMC3940601

[38] Zhang S Li X Wu J Coin L O'Brien J Hai F . Molecular methods for pathogenic bacteria detection and recent advances in wastewater analysis. Water. (2021) 13:3551. doi: 10.3390/w13243551

[39] Pardo A Villasante A Romero J. Skin microbiota of salmonids: a procedure to examine active bacterial populations using an RNA-based approach. Appl Microbiol. (2023) 3:485–92. doi: 10.3390/applmicrobiol3020034

[40] Olivares J Marshall SH. Determination of minimal concentration of *Piscirickettsia salmonis* in water columns to establish a fallowing period in salmon farms. J Fish Dis. (2010) 33: 261-266. doi: 10.1111/j.1365-2761.2009.01119.x 20088869

[41] Lannan CN Fryer JL. Extracellular survival of *Piscirickettsia salmonis*. *J Fish Dis*. (1994) 17: 545-548. doi: 10.1111/j.1365-2761.1994.tb00251.x

[42] Dettleff P Bravo C Patel A Martinez V. Patterns of *Piscirickettsia salmonis* load in susceptible and resistant families of *Salmo salar*. *Fish Shellfish Immunol*. (2015) 45:67–71. doi: 10.1016/j.fsi.2015.03.039 25862974

[43] Xue X Caballero-Solares A Hall JR Umasuthan N Kumar S Jakob E . Transcriptome profiling of Atlantic salmon (*Salmo salar*) parr with higher and lower pathogen loads following *Piscirickettsia salmonis* infection. Front Immunol. (2021) 12:789465. doi: 10.3389/fimmu.2021.789465 35035387 PMC8758579

[44] Pérez-Stuardo D Espinoza A Tapia S Morales-Reyes J Barrientos C Vallejos-Vidal E . Non-specific antibodies induce lysosomal activation in Atlantic salmon macrophages infected by *Piscirickettsia salmonis*. *Front Immunol*. (2020) 11:544718. doi: 10.3389/fimmu.2020.544718 33281810 PMC7688784

[45] Valdebenito EA Azúa M Silva-Sarmiento D Ojeda N Mercado L Valenzuela CA. Immune-like response and autophagy modulation in gill epithelial cells of rainbow trout challenged with *Piscirickettsia salmonis*. *Fish Shellfish Immunol*. (2025) 167:110878. doi: 10.1016/j.fsi.2025.110878 40946938

[46] Subramanian S MacKinnon SL Ross NW A. comparative study on innate immune parameters in the epidermal mucus of various fish species. Comp Biochem Physiol B Biochem Mol Biol. (2007) 148:256–63. doi: 10.1016/j.cbpb.2007.06.003 17618153

[47] Yu Y Wang Q Huang Z Ding L Xu Z. Immunoglobulins, mucosal immunity and vaccination in teleost fish. Front Immunol. (2020) 11:567941. doi: 10.3389/fimmu.2020.567941 33123139 PMC7566178

[48] Smith PA Contreras JR Rojas ME Guajardo A Díaz S Carbonero A. Infectivity of *Piscirickettsia salmonis* in immersion-bath exposed rainbow trout *Oncorhynchus mykiss* (Walbaum) fry. J Fish Dis. (2015) 38: 765-770. doi: 10.1111/jfd.12288 25168060

[49] Rozas-Serri M Peña A Maldonado L. Gene expression associated with immune response in Atlantic salmon head-kidney vaccinated with inactivated whole-cell bacterin of *Piscirickettsia salmonis* and pathogenic isolates. Fish Shellfish Immunol. (2019) 93:789–95. doi: 10.1016/j.fsi.2019.08.031 31419537

[50] Takano T Hwang SD Kondo H Hirono I Aoki T Sano M. Evidence of molecular toll-like receptor mechanisms in teleosts. Fish Pathol. (2010) 45:1–16. doi: 10.3147/jsfp.45.1

[51] Wu S Huang J Li Y Lei M Zhao L Liu Z. Integrated analysis of immune parameters, miRNA-mRNA interaction, and immune genes expression in the liver of rainbow trout following infectious hematopoietic necrosis virus infection. Front Immunol. (2022) 13:970321. doi: 10.3389/fimmu.2022.970321 36119061 PMC9479325

[52] Rodriguez MF Wiens GD Purcell MK Palti Y. Characterization of Toll-like receptor 3 gene in rainbow trout (*Oncorhynchus mykiss*). Immunogenetics. (2005) 57:510–9. doi: 10.1007/s00251-005-0013-1 16086174

[53] Brietzke A Korytár T Jaros J Köllner B Goldammer T Seyfert HM. Aeromonas salmonicida infection only moderately regulates expression of factors contributing to Toll-like receptor signaling but massively activates the cellular and humoral branches of innate immunity in rainbow trout (*Oncorhynchus mykiss*). J Immunol Res. (2015) 2015:901015. doi: 10.1155/2015/901015 PMC4525466 26266270

[54] Wang P Zhao C Wang C Fan S Yan L Qiu L. TLR3 gene in Japanese sea perch (*Lateolabrax japonicus*): molecular cloning, characterization and expression analysis after bacterial infection. Fish Shellfish Immunol. (2018) 76:347–54. doi: 10.1016/j.fsi.2018.01.013 29337246

[55] Sorini C Cardoso RF Gagliani N Villablanca EJ. Commensal bacteria-specific CD4+ T cell responses in health and disease. Front Immunol. (2018) 9:2667. doi: 10.3389/fimmu.2018.02667 30524431 PMC6256970

[56] Hino K Nakamura O Yoshiura Y Suetake H Suzuki Y Watanabe T . induces the growth of thymocytes in rainbow trout. Dev Comp Immunol. (2006) 30:639–47. doi: 10.1016/j.dci.2005.09.005 16368140

[57] Raida MK Buchmann K. Innate immune response in rainbow trout (*Oncorhynchus mykiss*) against primary and secondary infections with *Yersinia ruckeri* O1. Dev Comp Immunol. (2009) 33:35–45. doi: 10.1016/j.dci.2008.07.001 18760303

[58] Fajardo C Santos P Passos R Vaz M Azeredo R Machado M . Functional and molecular immune response of rainbow trout (*Oncorhynchus mykiss*) following challenge with *Yersinia ruckeri*. *Int J Mol Sci*. (2022) 23:3096. doi: 10.3390/ijms23063096 35328519 PMC8948951

[59] Teles M MacKenzie S Boltana S Callol A Tort L. Gene expression and TNF-alpha secretion profile in rainbow trout macrophages following exposures to copper and bacterial lipopolysaccharide. Fish Shellfish Immunol. (2011) 30:340–6. doi: 10.1016/j.fsi.2010.11.006 21078395

[60] Talbot AT Pottinger TG Smith TJ Cairns MT. Acute phase gene expression in rainbow trout (*Oncorhynchus mykiss*) after exposure to a confinement stressor: a comparison of pooled and individual data. Fish Shellfish Immunol. (2009) 27:309–17. doi: 10.1016/j.fsi.2009.05.016 19501170

