# Supplementary material for: Gene expression patterns of immune markers in rainbow trout during the early stages of seawater infections with Piscirickettsia salmonis
Source: Front Vet Sci. 2025 Nov 17;12:1660383. doi: 10.3389/fvets.2025.1660383 (PMC12667184; doi:10.3389/fvets.2025.1660383)
Supplement: Supplementary file 1 [file Data_Sheet_1.pdf]

Supplementary Material

Gene expression patterns of immune markers in rainbow trout during the early stages of seawater infections with *Piscirickettsia salmonis*

Héctor A. Levipan, Hernán Wicki, Fernanda Barrios-Henríquez, Francisco Pozo-Solar, Rute Irgang, Ruben Avendaño-Herrera

*** Correspondence to:** Héctor A. Levipan. hector.levipan@upla.cl / Ruben Avendaño-Herrera, ravendano@unab.cl

Supplementary Figures:

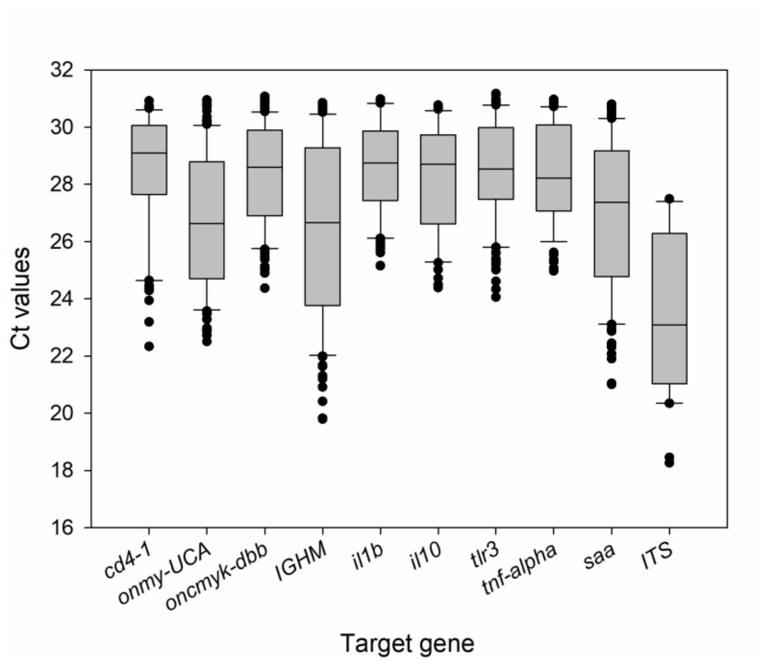

Supplementary Figure 1. RT-qPCR Ct values for target genes. Cycle threshold (Ct) values for each gene across all samples. Each box represents the interquartile interval range, which contains the central 50% of the data. The horizontal line within each box indicated the median Ct values..

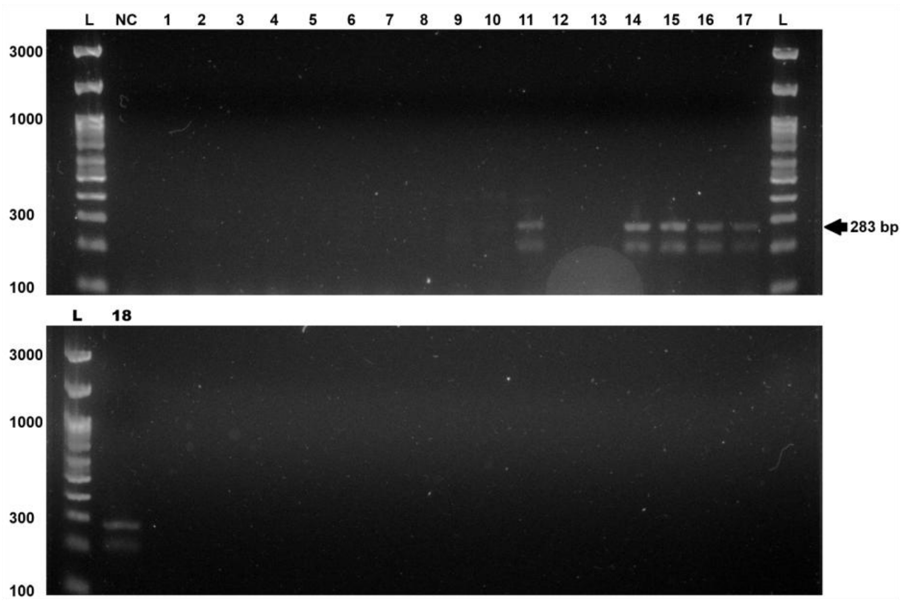

Supplementary Figure 2. Agarose gel electrophoresis (1.5%) of *P. salmonis* ITS amplicons obtained from cDNA templates using the RTS1-RTS4 primer set. L: AccuRuler molecular weight marker (100 bp Plus DNA RTU Ladder). NC = PCR negative control. Lines 1-3: analysis of the liver, spleen, and head kidney from a randomly collected fish in the pre-challenge condition. Lines 4-6: analysis of the liver, spleen, and head kidney from a randomly collected surviving fish in the negative control group after 30 days. Lines 7 and 8: analysis of the spleen and head kidney from a randomly collected surviving fish in the sterile SRS medium group after 30 days. Lines 9-14: LF89^{T}-caused mortalities at 30 dpi; mortality 1 (lines 9-11: liver, spleen, and head kidney) and 2 (lines 12-14: liver, spleen, and head kidney). Lines 15 and 16: analysis of the spleen and head kidney for a Psal-103-caused mortality at 30 dpi. Lines 17 and 18: analysis of the spleen and head kidney for a Psal-104-caused mortality at 30 dpi. The primer pair (refer to 26 in the manuscript's bibliography) amplified bands of expected size (283 bp) along with a shorter, likely non-specific product from cDNA templates. Nevertheless, the results were suitable for detection purposes.

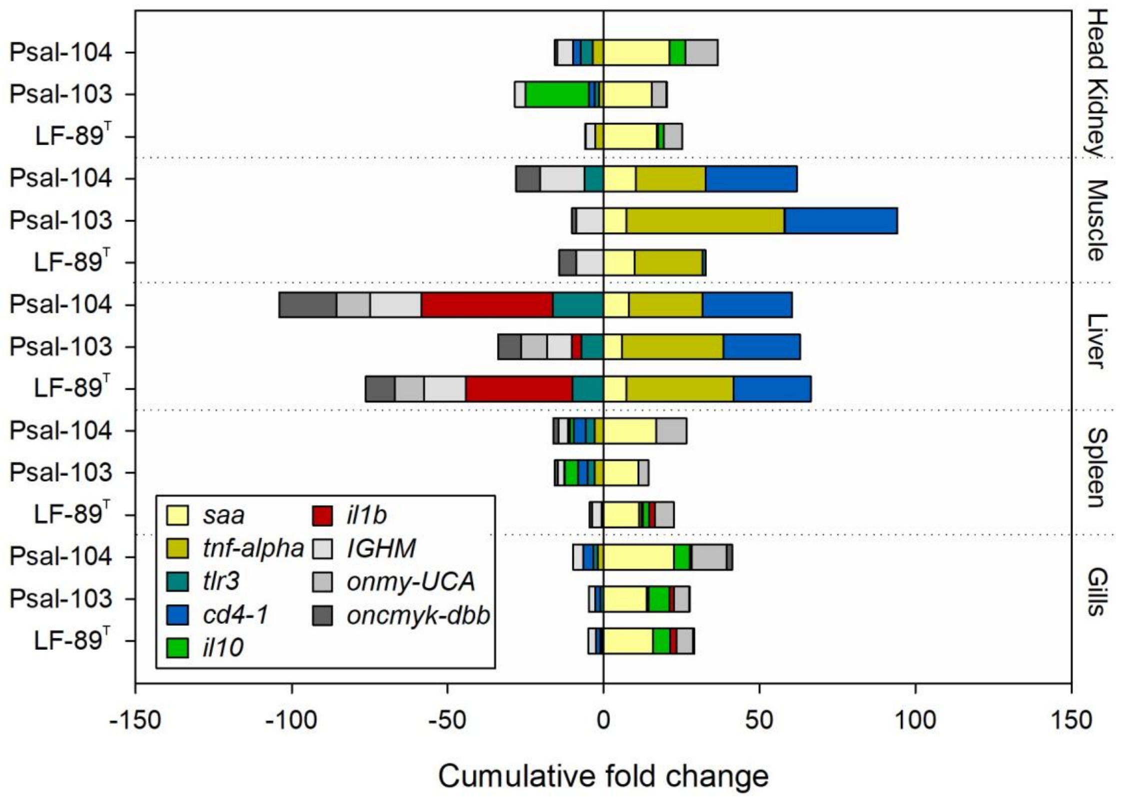

Supplementary Figure 3. Stacked bar plot showing cumulative fold changes over time (15 and 30 dpi) for target genes. The cumulative fold change was calculated using log_2-FC in gene expression between different infectious treatments (involving different *P. salmonis* genotypes and tissues) and the pre-challenge condition.
